# Phagocytic response to fully controlled plural stimulation of antigens on macrophage using on-chip microcultivation system

**DOI:** 10.1186/1477-3155-4-7

**Published:** 2006-08-16

**Authors:** Kazunori Matsumura, Kazuki Orita, Yuichi Wakamoto, Kenji Yasuda

**Affiliations:** 1 Department of Life Sciences, Graduate school of Arts and Sciences, The University of Tokyo, 3-8-1 Komaba, Meguro, Tokyo 153-8902, Japan; 2 Division of Biosystems, Institute of Biomaterials and Bioengineering, Tokyo Medical and Dental University, 2-3-10 Kanda-Surugadai, Chiyoda-ku, Tokyo 101-0062, Japan

## Abstract

To understand the control mechanism of innate immune response in macrophages, a series of phagocytic responses to plural stimulation of antigens on identical cells was observed. Two zymosan particles, which were used as antigens, were put on different surfaces of a macrophage using optical tweezers in an on-chip single-cell cultivation system, which maintains isolated conditions of each macrophage during their cultivation. When the two zymosan particles were attached to the macrophage simultaneously, the macrophage responded and phagocytosed both of the antigens simultaneously. In contrast, when the second antigen was attached to the surface after the first phagocytosis had started, the macrophage did not respond to the second stimulation during the first phagocytosis; the second phagocytosis started only after the first process had finished. These results indicate that (i) phagocytosis in a macrophage is not an independent process when there are plural stimulations; (ii) the response of the macrophage to the second stimulation is related to the time" delay from the first stimulation. Stimulations that occur at short time intervals resulted in simultaneous phagocytosis, while a second stimulation that is delayed long enough might be neglected until the completion of the first phagocytic process.

## Background

Phagocytosis as an effector mechanism of the innate immune response could be triggered by attachment of antigens to the surface of macrophages. The protein-based understanding of the signal processing pathways of innate immunity to microorganisms like Toll-like receptors (TLR), nucleotide-binding oligomerization domain (NOD) proteins, and myeloid differentiation primary-response protein 88 (MyD88) families for pathogen-associated molecular patterns (PAMs), has contributed to the development of therapeutics for human immune diseases [1,2]. However, it is still hard to explain the variability of responses caused by a lack of knowledge of the modulation mechanism of the immune response of single macrophages against multiple antigen stimulations. In other words, we still do not know whether signal processing can work simultaneously and independently against a plurality of antigen stimulations in different places on the surface of a single macrophage.

To understand the mechanism of complex signal processing that occurs in phagocytosis when there are multiple stimulations to macrophages, we need to give a series of fully controlled stimulations to an isolated single macrophage step-by-step under isolated circumstances. This is because with conventional group-based cultivation in a dish, stimulation of antigens to the target macrophage is usually done in an uncontrolled probabilistic way. Moreover, the physical contact with other macrophages might also influence the phagocytic response of macrophages.

In this paper, we report the time course of phagocytosis of an isolated single macrophage against a plurality of stimulations with antigens. In the experiment, to prevent the effects of unexpected factors, we used our on-chip single-cell cultivation system to give fully controlled stimulations to the isolated macrophage, and we then measured its response to those stimulations.

## Methods

### On-chip single-cell cultivation system

Previously, we developed an on-chip single-cell cultivation system exploiting the microfabrication technique and optical trapping. We applied this system to measure the adaptation process of isolated *E. coli*, to measure the size- and pattern-dependency of the community effect of cardiac myocytes, and to measure the response of a single-cell-based neural network pattern on a chip [3-9]. The system enables us to keep the condition around the cells constant under isolated conditions, and we can also physically add or remove other microorganisms by use of optical trapping. Individual cells in microchambers can be observed with a spatial resolution of 0.2 μm by phase-contrast/fluorescence microscopy.

**Figure 1:**
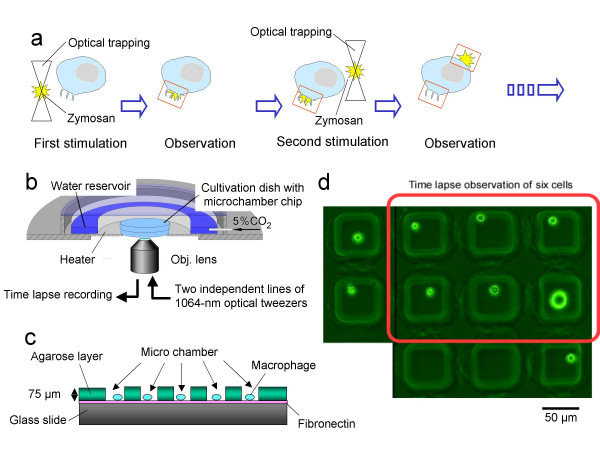
On-chip single cell cultivation system. Schematic drawings of experimental procedure (a), microcultivation system with microchamber chip (b), cross-sectional view of microchamber chip (c), top view of micrograph of microchamber array and macrophages (d).

To measure the macrophage response, as illustrated in Fig. 1(a), the protocol was as follows: the first antigen (zymosan) was trapped by optical tweezers and applied to stimulate the macrophage; then the second antigen was trapped by another optical tweezers and it was applied to stimulate the other side of the same macrophage; after the stimulation, a change in the shape was observed by time lapse recording over a long period. Figure 1(b) depicts the set-up of the on-chip single cell cultivation system. Macrophages were cultivated in the microchamber chip set in the cultivation dish. Temperature, humidity, and other conditions of the dish were completely controlled on the stage of the microscope during cultivation for long-term time lapse observation with a charge-coupled device (CCD) camera (CS220, Olympus) connected with the video-capture computer system. Two independent 1064-nm wavelength infrared optical tweezers (max. 1.5 W; PYL-1-1064-M, IPG Photonics, Oxford, MA, USA) were arranged in this system to handle two antigens simultaneously. Figure 1(c) shows a cross-sectional view of the microchamber's design fabricated in the cultivation chip, on which a thin layer of fibronectin and 75-μm-thick microstructures in an agarose layer were fabricated. To coat fibronectin (Wako Pure Chemical Industries, Osaka, Japan) on the washed glass slide (Asahi techno glass Corp., Chiba, Japan), 1 ml of 6-μg/μl fibronectin solution (in phosphate buffered saline; PBS) was deposited. The device was then incubated for 2 h, rinsed with PBS, filled with 3 ml of Macrophage-SFM medium, and placed in a 5% CO_2 _incubator at 37°C. To form the microstructure of agarose on the chip, a 1480-nm focused infrared laser beam was irradiated to melt a portion of the agarose layer. Figure 1(d) shows the top-view of the microchambers used in this experiment. Macrophages were cultivated in each microchamber under isolated conditions.

### Sample preparation and cultivation

Alveolar macrophages were isolated from five-week-old male CBA mice (Charles River Laboratories, Inc., Wilmington, MA). Immediately after sacrificing the animals by dislocation of the spine, their lungs were washed with 1 ml of Macrophage-Serum-Free medium (SFM) (Invitrogen, Carlsbad, CA). The cell suspensions (1 × 10^2 ^cells/ml) were plated on a fibronectin-coated microchamber array and incubated at 37°C in a 5% CO_2 _incubator. After incubation for 2 h, other non-adherent cells like erythrocytes were removed by washing. Then the dish was moved into the on-chip single-cell cultivation system. Zymosan particles (Molecular Probes, Eugene, OR) were reconstituted in a Macrophage-SFM medium and vortexed vigorously. To stimulate cells, 5 μl of 100-particles/μl zymosan resuspended solution were applied to the chip. During the on-chip cultivation we recorded changes in the surface shape of the macrophage, and we defined the starting time of phagocytosis to be when the surface shape of the macrophage at the point of zymosan attachment started to show specific changes.

## Results and discussion

**Figure 2:**
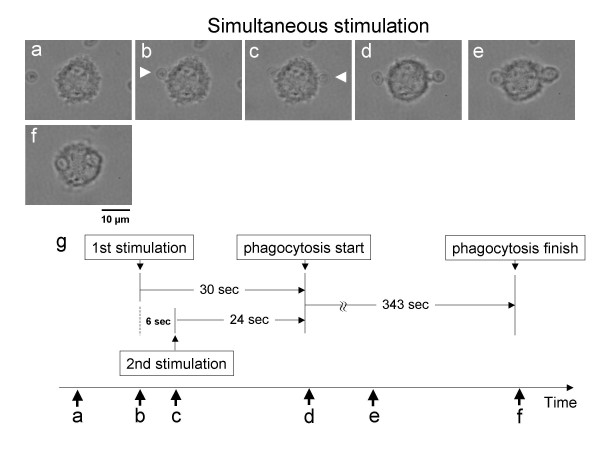
Time course of simultaneous stimulation. Micrographs before first stimulation (a), after first stimulation (b), after second stimulation (c), after phagocytosis started (d, e), and after phagocytosis was complete (f). Schematic explanation of time-course of simultaneous stimulation (g). White arrows in micrographs indicate the position of zymosan.

First, after we started cultivation on the chip, we simultaneously stimulated the isolated macrophage with two zymosan particles from opposite sides using two optical tweezers, as shown in Fig. 2. The two zymosan particles were attached to the macrophage within 6 s of each other (Fig. 2(d)) from the opposite direction. Phagocytosis started within 30 s on both sides, and both zymosan particles were phagocytosed simultaneously. The process of phagocytosis proceeded in the same manner and finished at almost the same time (343 s from the start). Six more experiments produced the same results: when the second stimulation occurred within 10 s of the first stimulation, simultaneous phagocytosis occurred.

**Figure 3:**
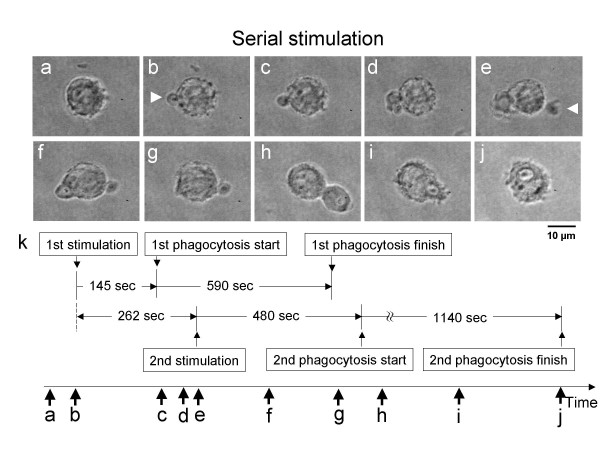
Time course of serial stimulation. Micrographs before stimulation (a), after first stimulation (b), after first phagocytosis started (c, d), after second stimulation started (e, f), after first phagocytosis was complete (g), second phagocytosis started (h, i), and after second phagocytosis was complete (j). Schematic explanation of time-course of series stimulation (k). White arrows in micrographs indicate the position of zymosan.

Next, we stimulated the isolated macrophage with two zymosan particles with different timing (Fig. 3). Just as in the previous experiment, we first stimulated one side of the macrophage with a zymosan particle using optical tweezers (Fig. 3(a–c)). Just 117 s after confirming the start of phagocytosis in the first attachment (Fig. 3(d)), we attached the second zymosan particle to the surface of the macrophage (Fig. 3(e)). Then, as shown in Fig. 3(e–g), even though the second zymosan was attached to the surface of the macrophage, phagocytosis did not start until the first phagocytosis process was finished (Fig. 3(h)). It should be noted that the required time to start the second phagocytosis process was less than 10 s after completion of the first process. Moreover, the time to complete phagocytosis for the first stimulation was about 590 s, whereas it took 1140 s for the second stimulation – about twice as long. When the second stimulation occurred more than 90 s after the first stimulation, the same delayed response of the second phagocytosis was observed in all four subsequent experiments.

**Figure 4:**
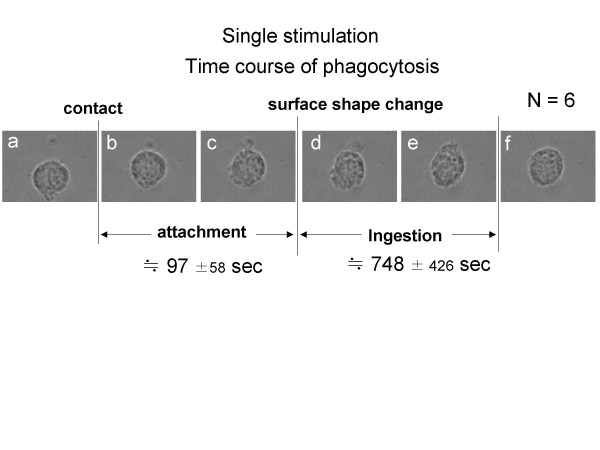
Time course of single stimulation. Micrographs before stimulation (a), after first stimulation (b, c), after phagocytosis started (d, e), after phagocytosis was complete (f). The time in the figure is averaged result of 6 samples.

To confirm the magnitude of variability of phagocytosis, we also measured the process of phagocytosis of single cells in the case of a single stimulation of zymosan. Figure 4 shows one example of the phagocytic process. The averaged time (from six samples) for the start of phagocytosis after attachment was 97 s, and it was 748 s for the complete phagocytosis process.

The results indicate that the delay of the second stimulation can produce a different response in the second phagocytic process of the macrophage depending on the timing of the second stimulation. If the second antigen stimulation started within 10 s of the first stimulation, the response of the macrophage was simultaneous. In contrast, if the second stimulation was delayed more than 47 s after the first stimulation, the phagocytosis of the second stimulant did not start until after the first phagocytosis was finished. As the waiting time for the second phagocytosis (480 s in Fig. 3) was much longer than the variability of the starting time of phagocytosis (average 97 s, max. 155 s in Fig. 4), the delay in the process after the second stimulation was not due to the variability of phagocytosis, but was apparently due to neglect during the first process even though the cell had been stimulated by the second stimulant. The two different macrophage responses to two stimulations indicate that some mechanism exists to control the timing of phagocytosis in the event of multiple stimulations. This shows the potential for simultaneous phagocytosis from two zymosan particles in different areas on the macrophage, as shown in Fig. 2. It also indicates that the initial phagocytic process can prevent a subsequent phagocytic process from occurring during the first process.

One possible explanation is that there may be a gathering of receptors on the cell membrane to the first antigen, and this may cause a lack of ability to sense the second stimulation at the opposite side until those receptors are released from the first antigen. The same gathering phenomena of sensor proteins were reported in T-cell receptors [10-12]. If the movement of sensing proteins on the macrophage is the explanation for these differences in response, the sensor proteins should move faster than 1 μm/s (10 μm of movement for less than 10 s) to respond to the second antigen within 10 s after the first phagocytosis is finished (see Fig. 3). That is, sensor molecules should disperse from one side of the macrophage to the other (ca. 10 μm in diameter) within 10 s. This diffusion velocity is within the magnitude of free diffusion velocity of cell membrane proteins, 5–10 μm^2^/s. In contrast, recent studies found that diffusion rates of many transmembrane proteins in the cell membrane are much lower than those in artificial reconstituted membranes by a factor of as much as 10 to 100, because the transmembrane proteins are corralled, or they undergo hop diffusion [13,14]. From this viewpoint, the movement of the sensor proteins for phagocytosis appears to resemble free diffusion rather than anchored transmembrane proteins or hop diffusion transmembrane proteins.

In conclusion, we applied an on-chip single-cell cultivation system to measure plural stimulation of antigens on the surface of isolated macrophages and found that a delayed second stimulation might be neglected until the first phagocytosis was complete. This phenomenon indicates that the phagocytic system does not work independently of the condition of the other side of the cell.

## Authors' contributions

KM, KO, and YW carried out the microchamber design, cell preparation, single cell observation, image analysis and also drafted the manuscript. Their contributions were equal. KY conceived of the study and participated in its design and coordination. All authors (KO was represented by KY) read and approved the final manuscript.

## Note

Ethical Permission No. 42 (to Yasuda Lab., April 1 2005, to March 31, 2006) was obtained from The Ethical Permission Organization of Animal Experiments in the Graduate School of Arts and Sciences, The University of Tokyo.
